# CRP identifies homeostatic immune oscillations in cancer patients: a potential treatment targeting tool?

**DOI:** 10.1186/1479-5876-7-102

**Published:** 2009-11-30

**Authors:** Brendon J Coventry, Martin L Ashdown, Michael A Quinn, Svetomir N Markovic, Steven L Yatomi-Clarke, Andrew P Robinson

**Affiliations:** 1 Department of Surgery & Tumour Immunology Laboratory, University of Adelaide, Royal Adelaide Hospital, Adelaide, South Australia, 5000, Australia; 2 Faculty of Medicine, University of Melbourne, Parkville, Victoria, 3052, Australia; 3 Department of Obstetrics & Gynaecology, University of Melbourne, Royal Womens' Hospital, Parkville, Victoria, 3052, Australia; 4 Melanoma Study Group, Mayo Clinic Cancer Center, Rochester, Minnesota, 55905, USA; 5 Berbay Biosciences, West Preston, Victoria, 3072, Australia; 6 Department of Mathematics and Statistics, University of Melbourne, Parkville, Victoria, 3052, Australia

## Abstract

The search for a suitable biomarker which indicates immune system responses in cancer patients has been long and arduous, but a widely known biomarker has emerged as a potential candidate for this purpose. C-Reactive Protein (CRP) is an acute-phase plasma protein that can be used as a marker for activation of the immune system. The short plasma half-life and relatively robust and reliable response to inflammation, make CRP an ideal candidate marker for inflammation. The high- sensitivity test for CRP, termed Low-Reactive Protein (LRP, L-CRP or hs-CRP), measures very low levels of CRP more accurately, and is even more reliable than standard CRP for this purpose. Usually, static sampling of CRP has been used for clinical studies and these can predict disease presence or recurrence, notably for a number of cancers. We have used *frequent serial *L-CRP measurements across three clinical laboratories in two countries and for different advanced cancers, and have demonstrated similar, repeatable observations of a cyclical variation in CRP levels in these patients. We hypothesise that these L-CRP oscillations are part of a homeostatic immune response to advanced malignancy and have some preliminary data linking the timing of therapy to treatment success. This article reviews CRP, shows some of our data and advances the reasoning for the hypothesis that explains the CRP cycles in terms of homeostatic immune regulatory cycles. This knowledge might also open the way for improved timing of treatment(s) for improved clinical efficacy.

## C-Reactive Protein (CRP) as an Acute-Phase Marker

C-Reactive Protein (CRP) is an acute-phase plasma protein that can be used as a marker for activation of the immune system. Acute-phase plasma proteins comprise a range of proteins that rapidly change in concentration in the plasma in response to a variety of stimuli, most notably inflammation and tissue injury. This 'acute-phase response' is also seen with progression of some malignancies and alteration in activity of various diseases, such as multiple sclerosis, diabetes, cardiovascular events, inflammatory bowel disease, infection and some autoimmune disorders. The liver produces many of these acute-phase reactants. CRP can be regarded as a 'positive' acute-phase protein because it characteristically rises *directly *with increased disease activity. Some other acute-phase proteins are termed 'negative' acute-phase proteins because these respond inversely with increased disease activity. In healthy individuals, CRP is naturally very low and difficult to detect in the blood. Although, a diurnal variation was absent in a small study, a recent larger study has reported a peak at about 1500 hours each day, with a variation in CRP level attributed to the diurnal, seasonal, and processing effects of 1%, and only a very small change occurred during the menstrual cycle in females. CRP did not show any significant seasonal heterogeneity [1,2]. When inflammation occurs there is a rapid rise in CRP levels, usually proportional to the degree of immunological stimulation. When inflammation resolves the CRP rapidly falls. Collectively, these properties make CRP potentially useful as a marker of active inflammation in certain situations.

## Synthesis and Types of CRP

CRP is produced by the liver and by adipocytes in response to stress. It is a member of the pentraxin (annular pentameric disc-shaped) family of proteins, and is not related to C-peptide or protein C [3]. The CRP gene is located on chromosome one (1q21-q23) which encodes the CRP monomeric 224 residue protein [4], but naturally secreted CRP comprises two pentameric discs. Glycosylation of CRP occurs with sialic acid, glucose, galactose and mannose sugars. Differential glycosylation may occur with different sugar residues in different types of diseases. The glycosylation that occurs in a specific disease is usually similar in nature, but the pattern of glycosylation varies between different disease types [5]. This can confer some relative specificity for patients having a similar disease.

## Role of CRP

The physiological function for CRP in the immune system is as a non-specific opsonin attaching to and coating the surface of bacterial cell walls or to auto-antigens, to enhance phagocytosis for the destruction or inhibition of bacterial cells or for the neutralisation of auto-antigens, respectively. The opsonin is recognised through the Fcγ2 receptor on the surface of macrophages or by binding complement leading to the recognition and phagocytosis of damaged cells. It was originally described in the serum of patients with acute inflammation as a substance reacting with the C-polysaccharide of pneumococcus [6]. Local inflammatory cells (neutrophils and macrophages) secrete cytokines into the blood in response to injury, notably interleukins IL-1, IL-6 and IL-8, and TNFα. The cytokines, IL-6, IL-1 and TNF-α are inducers of CRP secretion from hepatocytes [7], and therefore CRP levels serve as a marker of inflammation and cytokine release.

## Regulation of CRP

CRP is termed 'acute-phase' because the time-course of the rise above normal levels is rapid within 6 hours, peaking at about 48 hours. The half-life of CRP is about 19 hours and relatively constant, so that levels fall sharply after initiation unless the plasma level is maintained high by continued CRP production in response to continued antigen exposure and inflammation. It therefore represents a good marker for disease activity, and to some degree, severity. However, although it is not specific for a single disease process, CRP can be utilised as a tool for monitoring immune activity in patients with a particular disease [3]. Interleukin-6 (IL6), produced predominantly by macrophages and adipocytes, induces rapid release of CRP. CRP rises up to 50,000 fold in acute inflammation, such as severe acute infection or trauma. In most situations, the factors controlling CRP release and regulation are essentially those controlling inflammation or tissue injury. It is therefore relatively tightly regulated depending on the presence and degree of inflammation, with typical rises and falls in plasma CRP levels, forming a characteristic homeostatic, oscillatory cycle when inflammation occurs.

## Measurement of CRP

CRP assays are usually internationally standardised to permit more accurate comparison between laboratories. Various analytical methods, such as ELISA, immunoturbidimetry, rapid immunodiffusion and visual agglutination, are available for CRP determination. CRP may be measured by either standard or high-sensitivity (HS) methods. The HS method can measure low levels of CRP more accurately, so it is often termed Low-Reactive Protein (LRP or L-CRP). L-CRP below 1 mg/L is typically too small to detect, as is often the case in normal individuals, with minimal diurnal variation [1,2].

## Diagnostic Use of CRP Levels

Few known factors directly interfere with the ability to produce CRP apart from liver failure. CRP can be used as a marker of acute inflammation, however, persistent CRP levels can be used to monitor the presence of on-going inflammation or disease activity. Serial measurement of CRP levels in the plasma is indicative of disease progression or the effectiveness of therapy. Inflammation and tissue injury are the classical broad initiation signals for CRP release through the IL-6 mechanism, however, more specifically, infection is a typical cause for CRP elevation. In general, viral infections tend to induce lower rises in CRP levels than bacterial infections. CRP also rises with vascular insufficiency and damage of most types, which includes acute myocardial injury or infarction, stroke and peripheral vascular compromise. Elevation of the CRP level has predictive value for an increased risk of an acute coronary event compared to very low CRP levels. Similar findings have been reported with associations between increased risk of diabetes and hypertension. CRP levels have also been used to predict cancer risk, detect cancer recurrence and determine prognosis [7-16].

## CRP and Cancer

Recent evidence has associated CRP elevation using static measurements with progression of melanoma, ovarian, colorectal and lung cancer, and CRP has been used to detect recurrence of cancer after surgery in certain situations [7-13]. Persistent elevation of CRP, using several measurements weeks or months apart, has also has been reported for the detection of the presence of colorectal cancer and independently associated with the increased risk of colorectal cancer in men [14], and overall cancer risk [15]. Interleukin-6 (IL-6) has been used for the diagnosis of colorectal cancer and CRP was directly associated with survival/prognosis [16], but has been less widely used and not yet used serially. IL-6 is more expensive, more liable to variability, has a very short half-life (103 +/- 27 minutes) and has been shown to be less reliable than high-sensitivity CRP. As yet, therefore, it and other biomarkers, offer no tangible benefit over CRP currently as an assay for tracking the immunological cycle.

## Identifying Immune Oscillatory Cycles in Advanced Cancer using L-CRP

**Figure 1:**
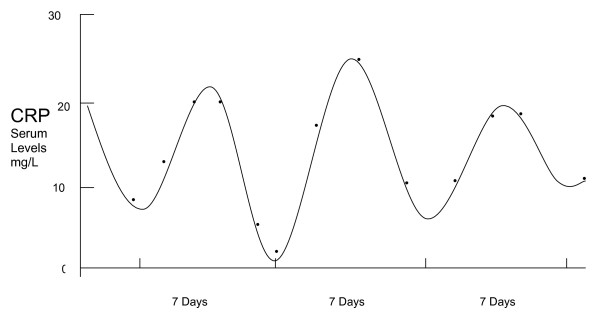
**CRP cycle in a patient with advanced melanoma**. Representative oscillation in L-CRP serum levels (y-axis; 0-30 mg/L) vs time in days (x-axis; bars show 7 days duration) in a patient with advanced melanoma, as also observed in other patients with advanced melanoma (Adelaide). From the serial CRP data-points a 'standard CRP curve' was mathematically derived.

**Figure 2:**
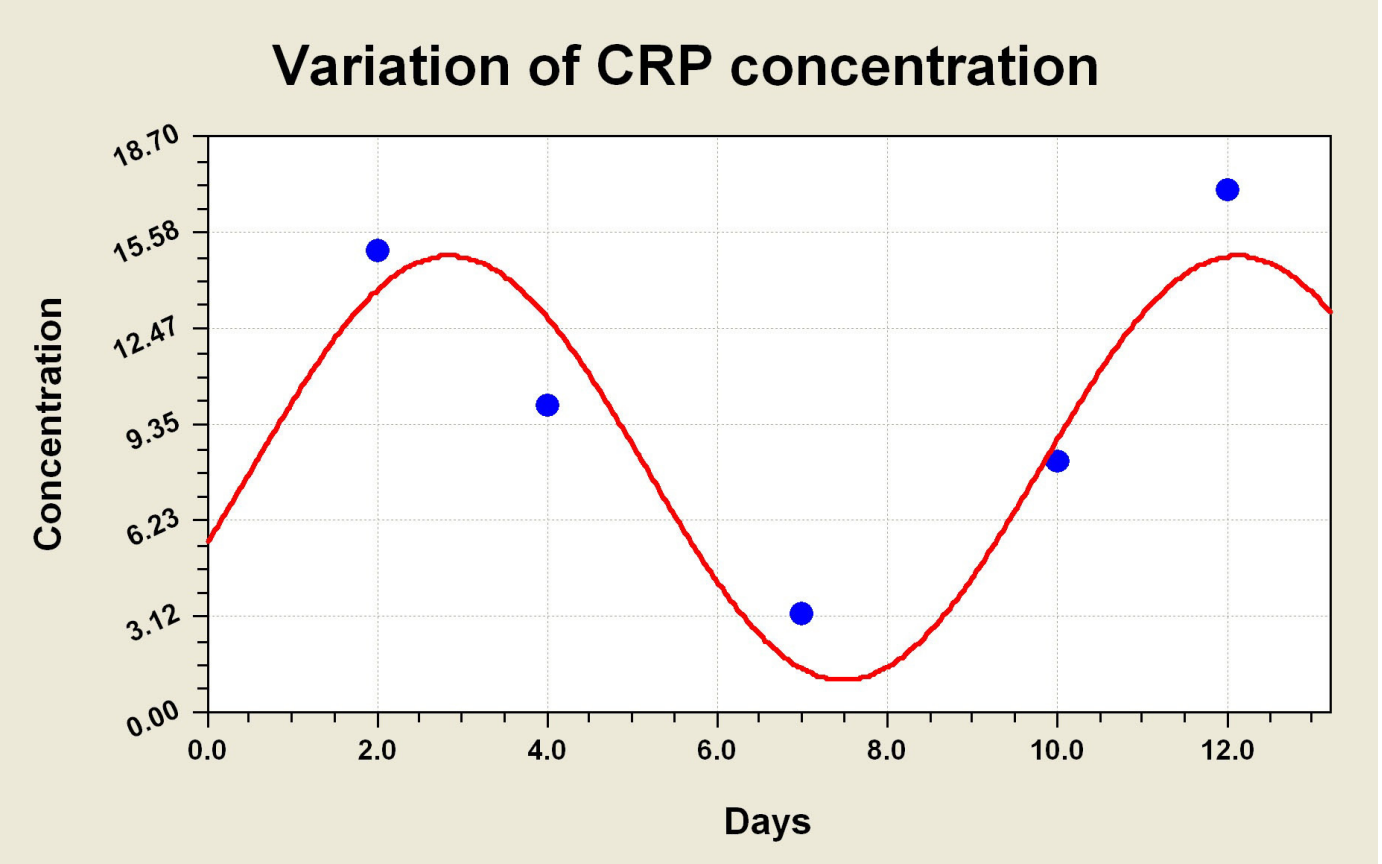
**CRP cycle in a patient with advanced melanoma**. A patient with advanced melanoma showing a similar L-CRP cycle to figure 1; CRP level vs days (Mayo, Rochester). From the serial CRP data-points a 'standard CRP curve' was mathematically derived.

**Figure 3:**
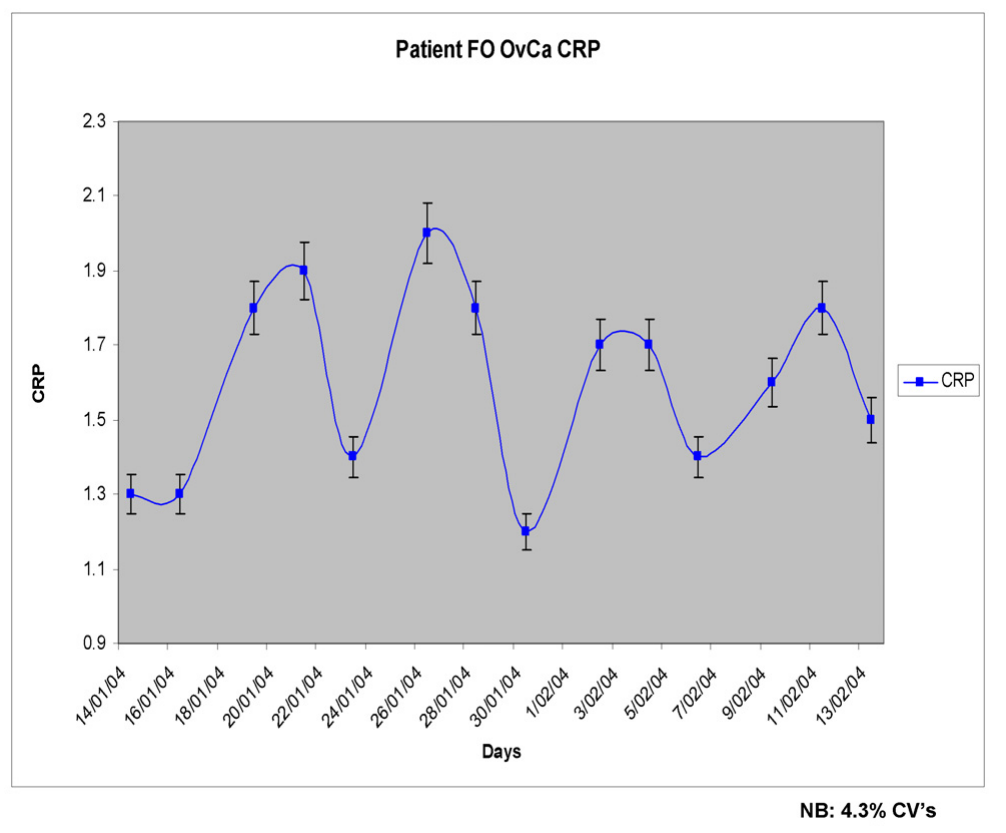
**CRP cycle in a patient with advanced ovarian carcinoma**. Measured oscillation in L-CRP levels vs time in days in a patient with advanced ovarian cancer (Melbourne). From the serial CRP data-points a 'standard CRP curve' was mathematically derived.

Single measurements of CRP or L-CRP have previously been used to correlate with the risk of certain cancers, prognosis or cancer recurrence, as mentioned above, and occasionally these have been repeated weeks or months apart to determine any persistence or trends in CRP levels. However, we have examined L-CRP in the serum of patients with advanced melanoma and ovarian cancer, *measured serially 1-2 days apart*, and identified an apparent 'cycle' in the CRP levels. Serial L-CRP measurements were plotted to rise and fall in a cyclical manner over time. These immune oscillations were dynamic in the cancer patients studied, revealing an apparent cycle, with a periodicity of approximately 6-7 days, in most situations. The amplitude appears to increase and decrease in response to the intensity of overall inflammation and disease activity. This is not dissimilar from previous work concerning haematopoiesis [17]. The observations might explain some of the clinical fluctuations in cancer growth and immune response activity, which is what led us to study more frequent measurement of CRP initially. Figures 1, 2 and 3 provide preliminary examples (clinical & statistical) of how the inflammation marker C-Reactive Protein (L-CRP) exhibits a regular homeostatic oscillation or cycle when measured serially (4 measurements; 1-2 days apart, and repeated) over time, in late-stage advanced cancer patients. The periodicity of 7 days for this cycle appears reasonably stable and reproducible amongst all of the patients (15 melanoma, 4 ovarian cancer, 1 bladder cancer and 1 multiple myeloma) so far examined, across three collaborative centres. These findings indicate some reproducibility and consistency amongst many patients with advanced cancer. The figures 1 to 3 show that the periodicity remains remarkably steady at around 7 days, irrespective of the amplitude of the CRP levels. The amplitude has been the main focus of previous cancer studies, principally because of the fact that close serial measurements have not been performed before, and the CRP levels have largely preoccupied attention because it has been (probably correctly) interpreted that these levels mirror disease activity.

Figures 1, 2 and 3 have relied on multiple serial measurements of L-CRP plotted against time to establish the individual 'CRP curve' for each patient over time. From the serial CRP data-points a 'standard CRP curve' was mathematically derived, which revealed a recurring or repeating curve every 7 days (trough to trough; or peak to peak). This 'standard CRP curve' has taken into account periodicity only, regardless of the individual amplitudes of CRP which may be subject to relatively high variability. The displayed data are from studies of single patients, and formal correlation between the CRP levels, cycles and clinical responses needs to be performed in larger numbers of patients before generalised conclusions can be applied.

## Defining the Position on the CRP Cycle

Serial L-CRP measurements were taken in the weeks around the time of each dose (vaccine or chemotherapy), and then used to identify the position on the oscillating 'standard CRP curve' where the dose had been given (regardless of CRP amplitude). This position was then plotted on the 'standard CRP curve' for each dose. In this way, we could determine where each dose lay at the time of administration with respect to the CRP cycle or curve (ie. lying in a trough, at a peak or in-between).

**Figure 4:**
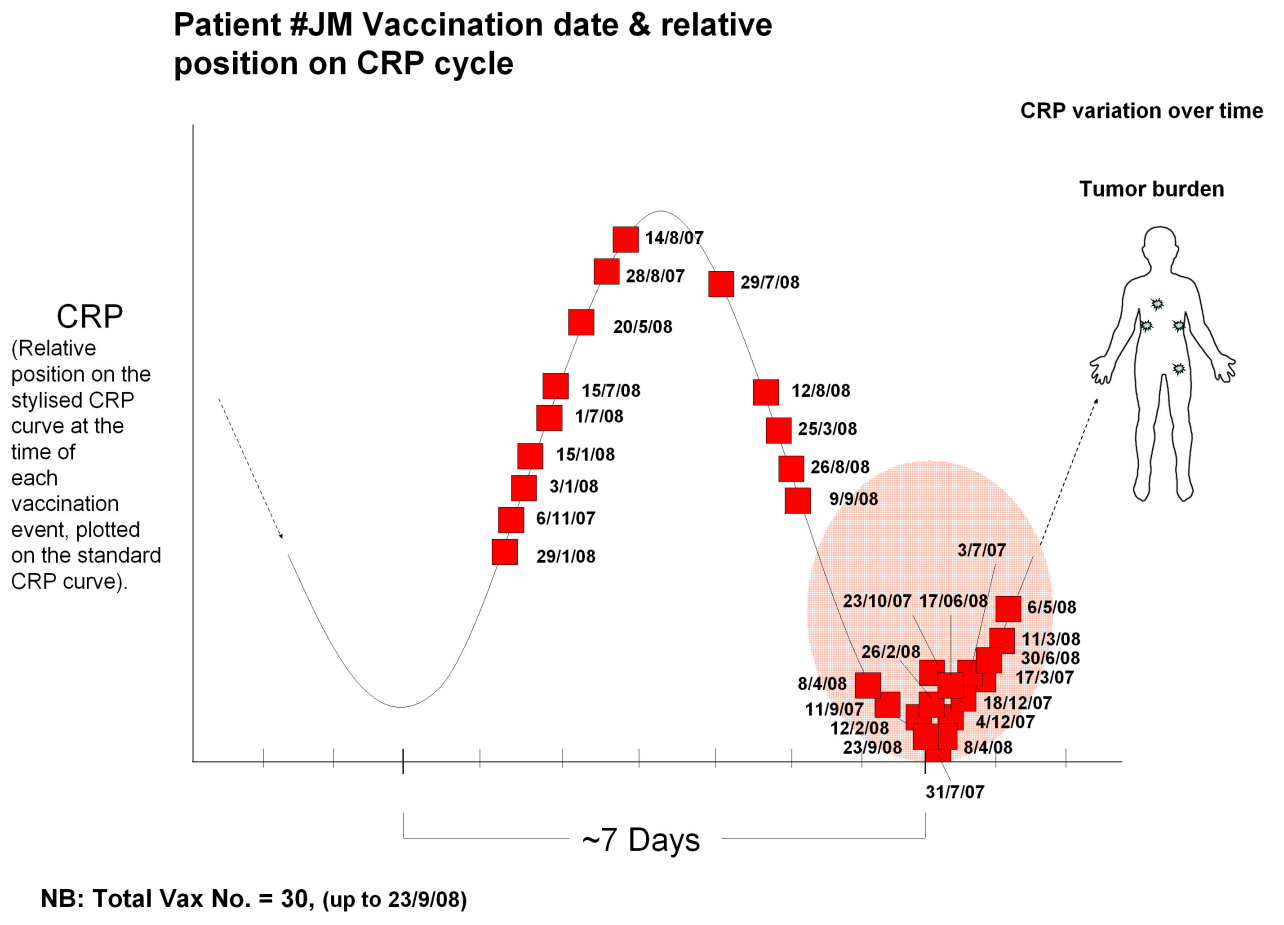
**Timing of Vaccinations with the CRP cycle in a patient with advanced melanoma**. Multiple fortnightly doses of vaccine in a patient with advanced melanoma showing the timing of each dose with respect to position (ie. trough, peak or in-between) on the L-CRP cycle (y-axis bar; L-CRP levels) vs time (x-axis; days; bars show 6-7 days duration), with repeated positions plotted for ease on the one 'stylised' CRP curve. Values are position on the CRP curve measured at the time of each vaccination, in the same patient (Adelaide).

**Figure 5:**
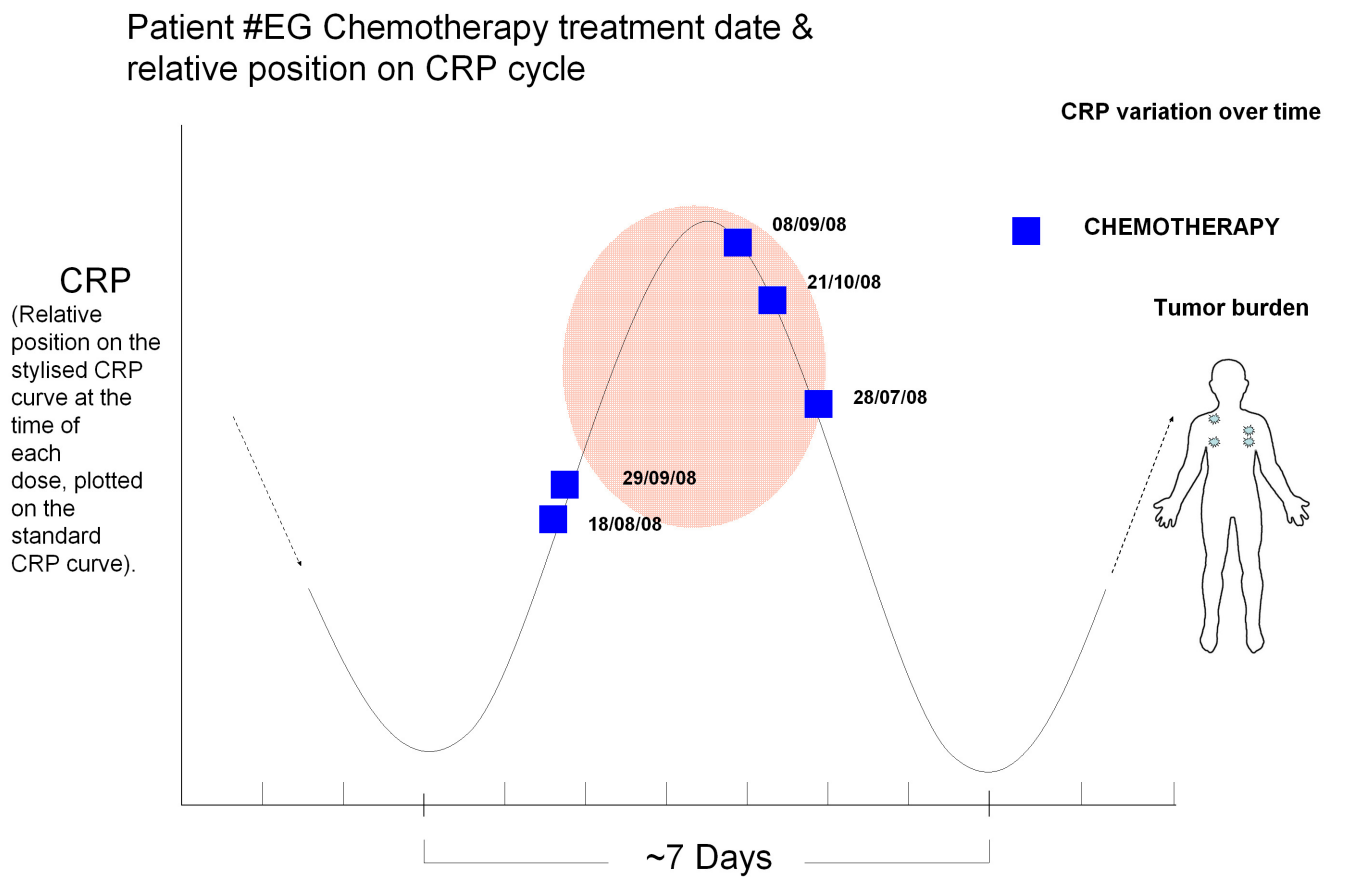
**Timing of chemotherapy with the CRP cycle in a patient with advanced melanoma**. Multiple doses of chemotherapy in a patient with advanced melanoma showing the timing of each dose with respect to position (ie. trough, peak or in-between) on the L-CRP cycle (y-axis bar; L-CRP levels) vs time (x-axis; days; bars show 6-7 days duration), with repeated positions plotted for ease on the one 'stylised' CRP curve. Values are position on the CRP curve measured at the time of each chemotherapy dose, in the same patient (Adelaide).

From the repeating or continuous CRP curve/cycle, a 'stylised CRP curve' using one cycle alone for representation was constructed, so that data from multiple repeating cycles could be shown on the one cycle. In reality, however, the CRP curve appears to be repeating as the immune system responds to the cancer in-vivo. Both Figures 4 and 5 (below) are based on a 'stylised' CRP curve, where we are only interested in where the dose occurred with respect to the CRP (inflammatory) cycle. Figures 4 and 5 show multiple doses of vaccine and chemotherapy, respectively, represented on a 'stylised CRP curve'.

## Possible Explanations: Regulatory Mechanisms of Immune Responses

A possible explanation of the observed L-CRP oscillation is that it might represent a rise with initiation and fall with termination of the immune response, which is indicative of a regulated anti-tumour immune response in the cancer patient, in a homeostatic fashion, similarly to inflammation from infection. This could best be explained by balance being maintained between effector responsiveness and tolerance [18], similarly to many endocrine on/off control mechanisms. Consequently, L-CRP may potentially act as a surrogate therapeutic biomarker of tumour specific T-effector and T-regulatory clonal expansion and activity. T-regulatory lymphocytes (T-regs) play a major role in attenuation of the T-effector response and animal data supports the concept that once tumour specific T-regs have been removed, tumour destruction and long-term survival can eventuate [19-22]. Currently, T-reg manipulation is being explored on a number of fronts, including with lymphodepletion [20]. Determining how to accurately target T-regs will undoubtedly be important in human therapeutic intervention. We hypothesise that successful, hitherto unrecognized, T-reg manipulation is already happening in the small percentage of cancer patients who get a complete response by virtue of spontaneous regression or with standard treatment. These are the patients who fortuitously receive therapy at the correct time-point (narrow window) in a repeating approximate 7-day cycle when T-regs are differentially and synchronously dividing, and are thus vulnerable to selective depletion with standard cytotoxic agents. This may also explain observations where cyclophosphamide acts as an inhibitor of T-reg activity [20]. Once regulatory circuits have been disrupted, the unmasked anti-tumour immune effector response can eradicate the tumour burden as has been reported in animal experiments [19]. It is also recognised that other explanations may exist and/or additional factors may be at play to explain or modulate the oscillatory cycles.

## CRP Oscillation and Other Diseases

Further clinical evidence for homeostatic immune oscillations is found in autoimmunity, especially associated with lymphodepletion or immunotherapy (eg. thyroiditis or vitiligo) [23], recovery from a viral illness (eg. shingles or upper respiratory infection) or bacterial infections, or with inflammatory bowel disease with repetitive cycles of worsening and recovery from disease. CRP levels have been used for monitoring disease activity in cardiovascular disease and diabetes [24-30], which emphasises the likely role of chronic inflammation in the aetiology [31,32].

## Immune Cycling and Cancer Treatments

Despite many attempts to stimulate the cancer patient's immune system for therapeutic benefit, results have been variable and often disappointing. Recent evidence suggests that an underlying persistent cyclical anti-tumour immune response is detectable in a number of tumour types, but is continuously being attenuated by the immune system's own regulatory mechanisms [33-35]. We propose that an understanding of this repeating immune cycle might be able to assist the clinician by pin-pointing recurring opportunities to selectively enhance T-effectors and/or deplete or inhibit T-reg cells, in a cycle specific manner, in the near future. Further well-controlled studies and work needs to be urgently done to substantiate the current observations.

## Examining the Hypothesis

### Vaccinations

We have examined this hypothesis by taking L-CRP measurements over the weeks surrounding the vaccination times of patients with advanced melanoma to determine the underlying L-CRP immune oscillatory cycle. Once this curve was established, we could then plot where on the L-CRP curve each vaccination had occurred. This allowed us to investigate the timing of vaccinations with respect to the CRP cycle, while examining the clinical responses. Since the periodicity of the L-CRP oscillatory cycle was consistent and recurrent, the results from multiple vaccinations could be plotted on a single representative 'standard CRP curve', showing the relative position on the CRP curve at the time that each vaccination was given. The current observations are demonstrated in Figure 4, which show that although vaccinations were randomly given over the CRP cycle, multiple vaccinations appeared clustered around the troughs of the L-CRP cycle. This patient had a good clinical response. At this time-point in the cycle T-effector cells would have been proliferating to produce the up-swing in CRP.

### Chemotherapy

We have investigated this hypothesis further by examining the timing of chemotherapy doses with respect to the L-CRP immune oscillatory cycle, in patients with advanced melanoma, while examining the clinical responses. The current observations are demonstrated in Figure 5, which shows that chemotherapy timing appeared clustered around the peaks of the L-CRP cycle. This patient responded well to chemotherapy. At this time-point in the cycle T-regulatory cells would have been proliferating to produce the down-swing in CRP.

## Conclusion and Future Directions

In summary, although CRP has been used as a static measurement and levels have been correlated with disease status and survival in cancer and other diseases, close multiple sequential measurements of CRP have essentially not been explored. CRP and especially L-CRP can be measured serially in the blood to demonstrate fluctuations in the levels of inflammation. Clinically, this CRP cycle appears to represent an underlying homeostatic oscillation in immunological reactivity in patients with advanced melanoma and ovarian cancer and possibly other malignancies. With this knowledge, we have explored the timing of vaccine and chemotherapy treatments in patients with regard to their clinical outcomes. What is emerging appears to be an association between the timing of delivery of the therapeutic agent(s) and improved outcome. This may open the possibility that in the future, vaccines and other biological agents may be able to be timed more specifically to maximise the immune effector response, to achieve an improved clinical outcome. Other strategies may be possible where inhibition of T-regs, for example by chemotherapy, radiotherapy or other treatments, could be more closely timed in an immune cycle-specific manner using the L-CRP oscillatory cycle. Some of the work using low-dose cyclophosphamide chemotherapy to deplete T-reg populations provides some evidence of this occurring by random application. On the basis of preliminary evidence, we hypothesise that the current random application of chemotherapy (or other immuno-cytotoxic therapy) with respect to the immune cycle might contribute to the poor clinical outcomes in the majority of late-stage cancer patients. Data is emerging from many human and animal studies that support this premise. It is therefore likely that better timing of administration of T-effector enhancing or T-reg depleting agents might be able to improve immune responses to break dominance of T-reg over T-effector cells, to achieve consistent improved longer-term survival benefits in cancer patients. Although it is too early to recommend this in clinical practice at present, we are currently actively exploring some of these exciting avenues of investigation.

## Competing interests

The authors declare that they have no competing interests, and all authors have read and approved the manuscript.

## Authors' contributions

BJC wrote and researched the manuscript; MLA contributed by original thought, research, reasoning, writing and modifications; MAQ and SNM contributed human data and manuscript comment; SLY-C and APR were involved in data analysis, modelling and manuscript comment.
